# A Heritable Antiviral RNAi Response Limits Orsay Virus Infection in *Caenorhabditis elegans* N2

**DOI:** 10.1371/journal.pone.0089760

**Published:** 2014-02-24

**Authors:** Mark G. Sterken, L. Basten Snoek, Kobus J. Bosman, Jikke Daamen, Joost A. G. Riksen, Jaap Bakker, Gorben P. Pijlman, Jan E. Kammenga

**Affiliations:** 1 Laboratory of Nematology, Wageningen University, Wageningen, The Netherlands; 2 Laboratory of Virology, Wageningen University, Wageningen, The Netherlands; Duke University Medical Center, United States of America

## Abstract

Orsay virus (OrV) is the first virus known to be able to complete a full infection cycle in the model nematode species *Caenorhabditis elegans*. OrV is transmitted horizontally and its infection is limited by antiviral RNA interference (RNAi). However, we have no insight into the kinetics of OrV replication in *C. elegans*. We developed an assay that infects worms in liquid, allowing precise monitoring of the infection. The assay revealed a dual role for the RNAi response in limiting Orsay virus infection in *C. elegans*. Firstly, it limits the progression of the initial infection at the step of recognition of dsRNA. Secondly, it provides an inherited protection against infection in the offspring. This establishes the heritable RNAi response as anti-viral mechanism during OrV infections in *C. elegans.* Our results further illustrate that the inheritance of the anti-viral response is important in controlling the infection in the canonical wild type Bristol N2. The OrV replication kinetics were established throughout the worm life-cycle, setting a standard for further quantitative assays with the OrV-*C. elegans* infection model.

## Introduction

The nematode *Caenorhabditis elegans* is an important model species for human biology. Research on this roundworm contributed to the understanding of cancer [1], aging [2], development [3], physiology [4] and the immune system [5]. Recently, a virus able to naturally infect this nematode was discovered, which infects the intestine inducing abnormal intestinal morphology [6], [7]. The causative agent, Orsay virus (OrV), was identified as a plus-strand RNA virus and putative member of the family *Nodaviridae*. The closest relatives to OrV are the co-discovered Santeuil virus [7] and Le Blanc virus [8], both of which infect *C. briggsae*. OrV persistently infects the *C. elegans* wild isolate JU1580 and is horizontally transmitted through the population in the laboratory [7].

The discovery of OrV coincides with increased sampling efforts and studies to expand the knowledge about natural variation in *C. elegans* [9]–[11]. Studying genotype-phenotype relations enhance our understanding of the ecological niche of *C. elegans* [10], [12], [13]. It is clear that this nematode thrives on decaying organic material. These short-lived and nutrient-rich environments mean that populations have the intrinsic property to grow fast [14]. It also places *C. elegans* in a complex web of inter-species interactions, where pathogens will often be encountered [10], [14], [15]. The variation in susceptibility to OrV in different genotypes is particularly interesting, as these can either be the result of adaptation from the side of the host (antiviral responses) or the virus (immune suppression). The availability of genetic variation in *C. elegans* can be combined with the powerful molecular tools also available for this model organism [16], [17].

The ability of OrV to complete a full replication cycle within its natural host *C. elegans* enables detailed studies on virus-host interactions [7]. This will lead to a better understanding of host specificity and identification of crucial genetic factors determining host susceptibility and/or resistance to viruses. In particular, the RNA interference (RNAi) response [18] plays a crucial role in the antiviral immune response of *C. elegans* [19]–[21]. Furthermore, the importance of antiviral RNAi is underscored by the fact that it is transmitted to the next generation, likely providing an advantage to the population as a whole [22]. Potent RNAi activity against OrV has also been observed in the canonical *C. elegans* N2 Bristol strain but less so in the natural isolate JU1580 [7]. This has been attributed to a mutation in the gene *drh-1* (a RIG-I like helicase), likely involved in the recognition of non-self RNA, including viral RNA [23]–[26]. However, nothing is known about the effects of mixed populations, prolonged virus exposure and the relative contribution of the trans-generationally inherited RNAi response. These unknown factors may also contribute to the progression of the infection.

To provide a deeper understanding of natural OrV infection, we set out to develop an infection procedure, which i) exposes *C. elegans* to OrV in liquid, ii) uses a defined viral dose, iii) times the exposure to infectious virus, and iv) enables larval stage-dependent infection kinetic studies. Using this procedure, we show the existence of genotype-dependent differences in the progression of OrV infection between *C. elegans* strains. In addition, a heritable RNAi response in the canonical N2 strain is identified as an important antiviral mechanism to convey resistance to its offspring.

## Materials and Methods

### C. elegans culturing

The strains N2, JU1580, WM29 (*rde-2*, ne221), and WM49 (*rde-4*, ne301) were kept at 12°C on 6 cm Petri dishes containing Nematode Growth Medium (NGM), seeded with *Escherichia coli* strain OP50 [27]. Before onset of the experiments, single worms were picked of each genotype and grown into a new population. They were grown at 20°C on 9 cm dishes. For synchronization the populations were bleached [28]. A virus free JU1580 population was created by bleaching an infected strain of JU1580 [7]. This uninfected strain was used as starting material for the experiments.

### Generating stocks of Orsay virus

Orsay virus was isolated from persistently infected nematodes of strain JU1580. The nematodes were kept at 16°C on NGM plates and transferred to new NGM plates every 14 days. Virus stocks were generated by isolation from *C. elegans* as previously described by [7]. PBS was used for isolating the virus from the worms. Virus stocks were flash frozen in liquid nitrogen and stored at –80°C. Before use in experiments the stocks were tested by infecting virus free JU1580 with different volumes (1, 10, 50, and 100 µL) and using an RT-PCR to confirm the establishment of viral replication. This yielded an estimate for the infectious dose, as no other assays are available.

### Infection procedure

Before infection the *C. elegans* strains were synchronized. Populations were grown at 20°C until the desired larval stage was reached: 20h for L1, 26h for L2, 40h for L3, or 48h for L4. To infect the synchronized population, the worms were collected by rinsing the plate with M9 buffer and centrifuged shortly to pellet the worms. Thereafter the M9 buffer was removed and 500 µL of infection solution (370 µL of M9, 30 µL of virus stock and 100 µL of OP50 in LB) or mock solution (400 µL of M9 and 100 µL of OP50 in LB) was added. The worms were incubated in infection solution for 1h in Eppendorf tubes at room temperature and regularly mixed to infect them with OrV. Next the worms were pelleted by centrifugation and the supernatant was removed. The worms were washed three times with 1 mL of M9 buffer to remove virus from the supernatant and thereafter plated on a fresh NGM plate containing OP50.

### RNA isolation and RT-qPCR

The RNA of infected *C. elegans* was isolated using the QIAGEN RNeasy Micro kit, following the prescribed protocol. cDNA was made using the SuperScript III kit from Invitrogen following the prescribed protocol with random hexanucleotides. Per RT-reaction 1 μg of isolated RNA was used. For the qPCR reaction the cDNA was diluted 1/50 and qPCR was performed with Absolute QPCR SYBR Green Fluorescein Mixes (Thermo scientific). Viral RNA was detected using two primer pairs, both annealing to the start of the RNA1 coding region (HM030970.1) (pOrV-RNA1.1F: 5′ATACTCTACGACCTTGTCGG 3′, pOrV-RNA1.1R: 5′CTCGGTTGATGTTCTTCCAG 3′, pOrV-RNA1.2F: 5′AACCAGGAAACACTACTCCG 3′, pOrV-RNA1.2R: 5′GTTGTGATATCGCTTGGTGG 3′). Two reference genes (Y37E3.8 and *rpl-6*) were selected based on stable expression, even during stress condition, in transcriptomics data generated by microarray (pY37E3.8F: 5′GCGTTTGTGGTCTCTTGTC 3′, pY37E3.8R: 5′CTCTGGGAGGAGTCCTTTTC 3′, pRPL6-F: 5′TGTCACTCTCCGCAAGAC 3′, pRPL6-R: 5′TGATCTTGTGTGGTCCAGTG 3′).

The primer pairs were designed for an optimal annealing temperature of 62°C [29], which was verified by testing the primers on a temperature gradient. Furthermore, specificity was checked by measuring the melting curve and efficiency by performing an RT-qPCR reaction on serially diluted template. The primer pairs do not generate unspecific products within 40 cycles and the measured efficiency was in-between 90% and 110% (100% being the product doubles every cycle).

### Data normalization

Between biological replicates the RT-qPCR data was checked using the reference genes, outliers per biological replicate were identified as having Ct values for the reference genes that fell outside µ +/– 2*σ of all the measurements. Outlier samples typically showed low RNA quality (e.g. partial degradation or contaminations). The expression of the reference genes was checked for genotype and larval stage effects. This to ensure that differences found were not the result from different reference gene expression (**Table S1**).

The data was transformed with



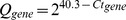



where Q is the expression of the gene and Ct is the measured Ct value of the gene. The number 40.3 indicates the level of the 5% highest Ct values in mock infected samples (based on 104 mock infected samples). Thereafter the viral measurements were normalized to the expression of the control genes



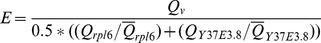



where E is the relative expression, Q is the transformed expression (v indicates either one of the viral genes, *rpl-6* and *Y37E3.8* are reference genes). All data was normalized together, to allow for direct comparison.

### Statistical analysis

Statistical tests were executed using custom written scripts in R (version x64 2.13.1, www.r-project.org). Pairwise testing was done using a two-sample independent t-test not assuming equal variances (Welch’s *t*-test), as provided by R. Testing over multiple samples was done by ANOVA, as provided by R.

Logistic curve fitting was performed using a non-linear model, fitting to a basic logistic curve with the function



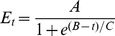



where E is the relative expression at time point t, A is the fitted upper asymptote, B is the fitted inflection point and C is the lower asymptote. To be able to fit the function, the relative expression had to be transformed so the lower asymptote approached 0, otherwise the SSlogis function [30] in R was not able to correctly estimate the inflection point. Confidence intervals were calculated using the predict.nls function of R. The goodness of fit was calculated as



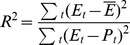



where R^2^ is the coefficient of determination, E is the relative expression at time point t, and P is the predicted value at time point t.

Multiple testing over the descriptive values obtained from the sigmoidal curve fitting was done using linear regression, by fitting the data to



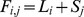



where F is the descriptive value obtained from the sigmoidal curve fitting, L is the larval stage i (L1, L2, L3, or L4), and S is the strain j (JU1580 or N2). When testing within one strain, the model was simplified by excluding the strain as an influence.

## Results

### Worm stage affects OrV infection development in *C. elegans* JU1580

**Figure 1:**
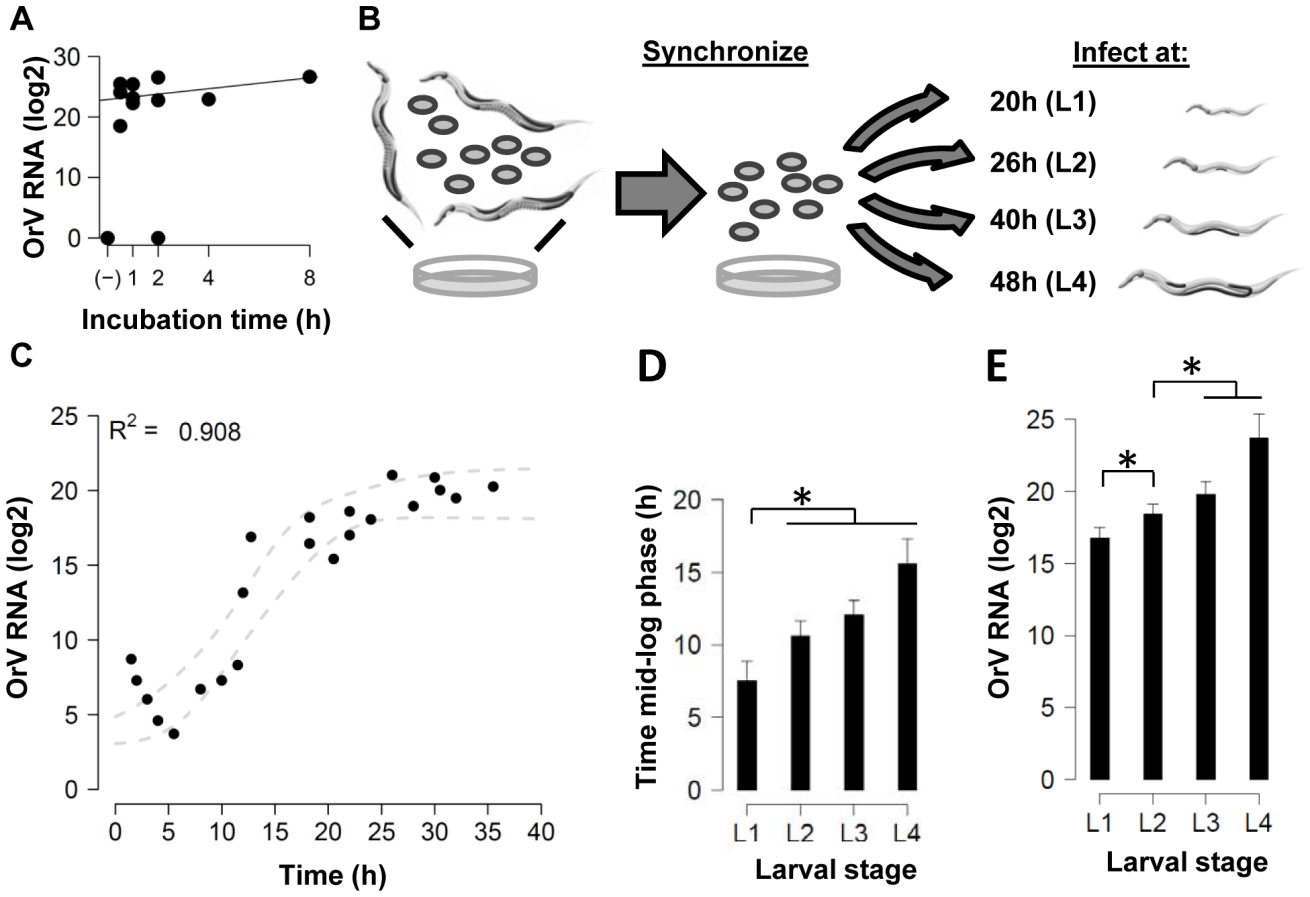
Infections in genotype JU1580. (**A**) JU1580 was exposed to 30 µL virus in solution for 0.5, 1, 2, 4 and 8h. The relative viral load measured by RT-qPCR 48h post infection is shown. There are no differences between the viral loads after different exposure times (ANOVA, P = 0.70). (**B**) The design of the experiments to measure the infection progress in different larval stages. First populations were synchronized by bleaching and subsequently grown until the desired larval stage was reached. At the indicated time points the larvae were infected by exposure to virus for 1h, after which the infection was allowed to develop and at different time points populations were isolated. (**C**) An example of the data, the outcome of the experiments performed in JU1580 when infected in the L3 stage (bullet points indicate sample points used for the fit). The dashed grey line indicates the SD around the fitted curve. (**D**) The time needed to reach the inflection point as determined by the curve fit is shown for JU1580. There is a significant difference between L1 and the other three larval stages (Two-sided *t*-test, P≤0.05). (**E**) The maximum viral load reached (the asymptote of the curve) in JU1580. Here the load of L1 versus the other three larval stages (Two-sided *t*-test, P≤0.05) and L2 versus the other larval stages (Two-sided *t*-test, P≤0.05) is significantly lower.

First, the exposure time needed to infect a population was established by incubation of JU1580 with OrV in liquid. Liquid infections have the following advantages: the dosage is the same for every worm, the infection is timed, it can be executed at a defined larval stage, and the worms can be washed to remove the non-internalized virions. A starved mixed stage population (mainly adults and L2 present) of JU1580 was exposed to OrV in infection solution for 0.5, 1, 2 and 4h. Relative viral loads were measured at 48 h post infection by quantitative (q)PCR. The results show that OrV infections can be established following 1h exposure (**Figure 1A**) and do not increase with longer exposure to the virus (ANOVA, P  =  0.70).

To investigate the relative susceptibility of *C. elegans* larval stages to OrV, virus infections were carried out in JU1580 synchronized populations of L1, L2, L3, and L4 larval stages (**Figure 1B**). An example of the data retrieved and a sigmoidal-curve fit for JU1580 infected at the L3 stage is shown in **Figure 1C** (curves for L1, L2 and L4 are shown in **Figure S1)**. For all stages the fit explained >80% of the variation. Within the first 3h after exposure, the viral levels decreased in all larval stages (see also **Figure S1**). Since the route of infection is oral uptake and the infection takes place in the intestine [7], this initial decrease most likely represents an overload of virus which is leaving the intestine but still measurable by qPCR. After the initial decrease a steady level (lag phase) is reached after which replication starts (log phase).The speed at which the infection develops is dependent on the larval stage (**Figure 1D**). In L1 larvae the inflection point (mid-log phase) is reached after 7.5h, whereas in the other stages it takes >10.5h (**Figure 1D**, significant difference, Two-sided *t*-test, P≤0.05). The maximum viral load is larval-stage dependent; significantly higher levels are reached in older larvae (**Figure 1E**). For instance, the difference in maximum viral load is 6.9 log2 units (>100-fold) between L1 versus L4 (Two-sided *t*-test, P≤0.05). In conclusion, in older larvae the OrV infection progresses slower, but reaches higher maximum viral loads.

### Comparative infections in *C. elegans* JU1580 and N2

**Figure 2:**
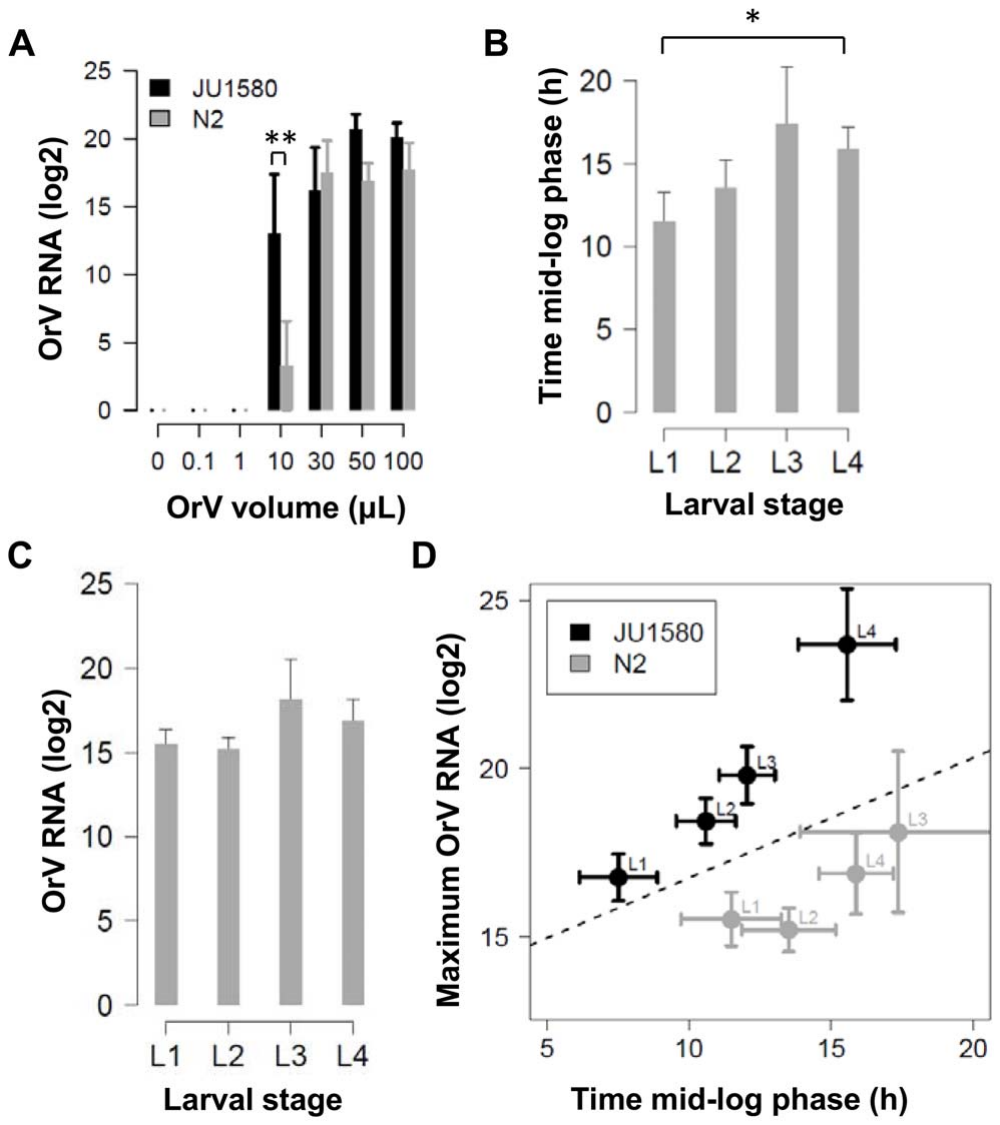
Infections in N2 compared with JU1580. (**A**) JU1580 and N2 were exposed to different amounts of virus (0, 0.10, 1.0, 10, 50, and 100 µL) in infection solution for 1h. The relative viral load measured by RT-qPCR 48h post infection is shown (+/– SEM), for 3 independent repeats in duplo per experiment (except 10, 50 and 100 µL, which were replicated 5 times in JU1580). JU1580 reaches maximum viral load after exposure to 10 µL (ANOVA, P>0.05), whereas N2 after exposure to 30 µL (ANOVA, P>0.05). (**B**) The time needed to reach the inflection point as determined by the curve fit is shown for N2. There is a small delay (approximately 4h) in reaching the mid-log phase between L3 and L4 versus L1 (Two-sided *t*-test, P≤0.05). (**C**) The maximum viral load reached (the asymptote of the curve) for N2. Here there are no larval-stage dependent effects (*t*-test, P>0.05). (**D**) A comparison of the values obtained for JU1580 (figure 2) and N2. The dashed line is a fitted linear function used to compare the maximum viral load and mid-log phase, showing that JU1580 has higher viral loads and/or a faster developing infection (ANOVA, P≤0.01).

We first investigated if the reported difference in susceptibility between the two wild types JU1580 and N2 [7] could be the result of differences between the dose-response relationship. The effect of viral dose was studied in the two genotypes, using an exposure time of 1h (**Figure 2A**). Since it was previously found that after long-term infection, *C. elegans* N2 strain displayed ∼100-fold lower viral levels compared to the wild strain JU1580 [7], we expected to see a comparable difference. Genotype JU1580 could be infected with a smaller dose to reach maximum viral load levels compared to N2. JU1580 could be infected by a dose as little as 10 µL of virus stock in 80% of the experiments, whereas N2 was only infected (at a very low level) in 33% of the experiments in which the dose-response was determined. However when more virus (>30 µL) was used, N2 was productively infected as well, and the differences became smaller, up to the point that JU1580 and N2 had comparable infection levels (ANOVA, P  =  0.09). Concluding, JU1580 and N2 are comparable in susceptibility when exposed to higher viral doses.

The dose-response experiments showed that there was no difference between maximum viral levels in JU1580 and N2, provided that larvae were exposed to sufficient amounts of OrV. This puts earlier findings [7] in a new perspective. Previously it was shown that N2 was less susceptible than JU1580 to OrV infection. The main differences between our experiments and the latter are the mode of infection (in liquid vs. on agar), the exposure time, and the population dynamics during the experiment. Viral loads in our experiments were measured up to 48h post infection as compared to 4-7d after exposure. In addition, our experiments predominantly involve virus infections within a single generation, in contrast to experiments on infected adults which in turn spread the infection to their offspring. Therefore, the apparent discrepancy might originate from these differences, exposure duration and/or re-infection.

To further compare the two genotypes our infection experiment as described in **Figure 1B** was also conducted in N2 in all four larval stages. The data obtained from the curve fits can be seen in **Figure 2B and C** (the separate curves showing all data points can be found in **Figure S1**). Like JU1580, the curve fits for N2 explained >80% of the variation. On average, the explained variation was a bit lower in N2 compared to JU1580. In N2 the mid-log phase of OrV infection is reached earlier in younger larvae and ∼4.5h faster in L1 versus L4 (two-sided *t*-test, P≤0.05). Maximum viral loads were similar in all N2 larval stages (two-sided *t*-test, P>0.05), in contrast to JU1580 for which there was an age-dependent increase in maximum viral load.

The initial infection kinetics of JU1580 and N2 were also compared (**Figure 2D**). It was found that, in general, the infection progresses at an equal pace in JU1580 and N2 (two-sided *t*-test, P≤0.05). The maximum viral load however, was significantly different between JU1580 and N2 for the larval stages L2 and L4 (P≤0.05). Overall, there was a trend that the maximum load in JU1580 is 10-fold higher (3.3 log2 units). However, these differences are not large enough to be the main source of the previously reported 100 fold difference in OrV infection levels between JU1580 and N2 populations [7]. The difference in maximum load between the two genotypes in the experiments described in this paper arises mostly in the L4 stage. In our experiment the worms were infected at an age of 48 hours, and the last hours of the experiment the first larvae are observed. Since we show that N2 and JU1580 are equally susceptible (not-previously exposed and at high viral dosages) and Félix *et al.* (2011) found a 100-fold difference in maximum viral load between the two after multiple generations we suspected that mechanisms, like the inheritance of an antiviral response, could play a role.

### The antiviral RNAi response suppresses the progression of infection in *C. elegans* N2

Since the antiviral RNAi response was shown to be an important factor in OrV replication [7], we investigated whether or not this response influences the initial infection. In parallel with the experiments described in **Figure 1B**, experiments were carried out in L3 larvae of *rde-2* and *rde-4* mutants in an N2 background. RDE-2 is involved in heritable silencing of RNA only and functions after initiation of the original RNAi response [31] and functions in concert with *mut-7* [32]. RDE-4 is involved in siRNA production from exogenous dsRNA, in concert with DCR-1. Mutants of *rde-4* are impaired in siRNA production and thus cannot initiate antiviral RNAi nor pass on to their offspring the heritable silencing signals [33]-[35]. Both the *rde-2* and *rde-4* mutants were shown to increase OrV infection to the level observed in JU1580 [7].

**Figure 3:**
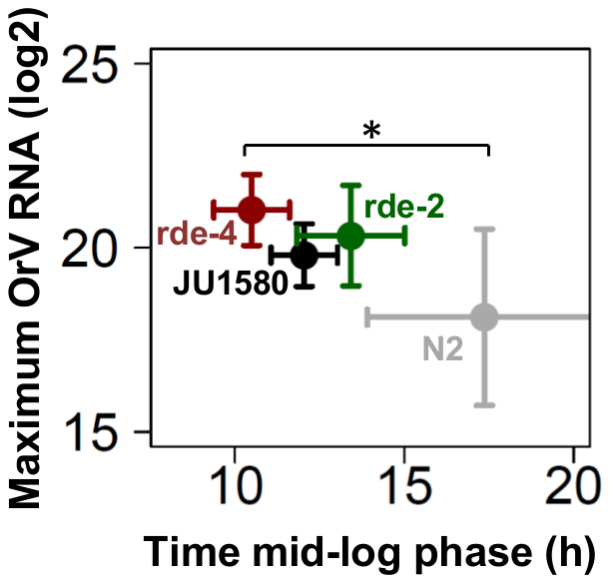
Infection assays in L3 of N2, JU1580, *rde-2* and *rde-4*. The maximum viral load reached and the time till the inflection point was reached is shown for four genotypes. The *rde-4* mutant reaches the mid-log phase significantly faster than N2 (Two-sided *t*-test, P≤0.05), whereas the other two genotypes are similar to N2 (Two-sided *t*-test, P>0.05). However, there is no significant difference between maximum viral load reached between the genotypes (Two-sided *t*-test, P>0.05).

Both *rde-2* and *rde-4* mutants displayed a JU1580 phenotype regarding maximum viral load reached (**Figure 3** **, Figure S1**), however compared to N2 these differences were not significant due to a larger variation in N2 (Two-sided *t*-test, P>0.05 in both cases). Importantly, the time until the mid-log phase was similar between the *rde-2* and N2 (Two-sided *t*-test, P>0.05). Whereas in the *rde-4* mutant the infection developed significantly faster than in N2 (Two-sided *t*-test, P≤0.05), showing that the initial recognition and cleavage of dsRNA is an important step in the progression of the infection, and therefore in the antiviral response in *C. elegans*. The effect on maximum viral load in both RNAi mutants points to JU1580 being compromised in provoking an effective antiviral RNAi response which is in line with recent findings [23], [24]. However, overall there is not a large difference between N2 and the genotypes impaired in the RNAi response: JU1580 and the RNAi mutants. This is in agreement with our previous findings, comparing only N2 and JU1580, which suggest that the main difference between N2 and JU1580 does not lie in the primary infection.

### Role of heritable RNAi in susceptibility to OrV infection

The combined results of the OrV infection experiments in N2 and JU1580 show that the initial infection development only contributes to a ∼10-fold difference in viral levels, which falls short to explain the difference in viral load between JU1580 and N2 reported previously [7]. Given that inheritance of RNAi is a well-studied phenomenon in *C. elegans* in general [18], as well as in the context of viral replication [22], the differences with previously published results [7] could be caused by an inheritable antiviral RNAi response.

**Figure 4:**
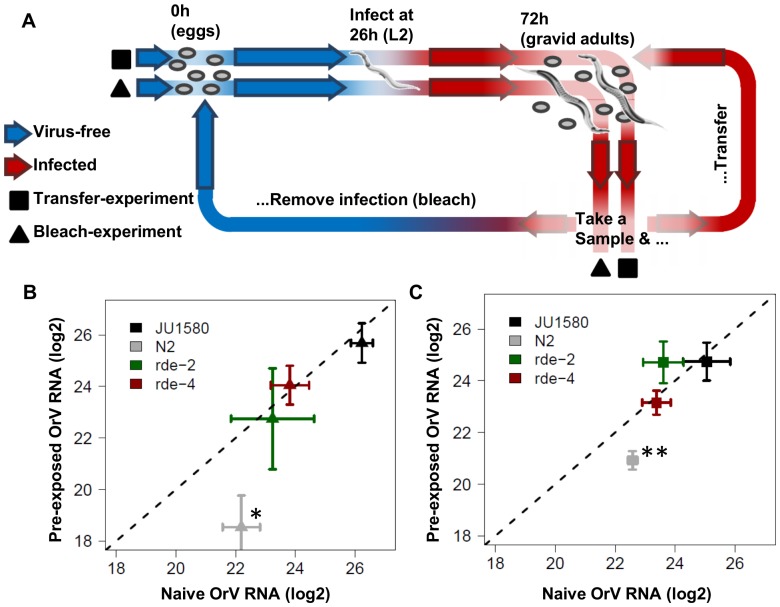
Trans-generational inheritance of antiviral RNAi repsonse. (**A**) Shows the outline of the experiment, worms were synchronized and 26h thereafter exposed to OrV. 72h past bleaching the worms have laid eggs, at this point the experiment progresses in two parts (indicated by the triangles and squares), either re-synchronized and re-infected populations (triangles) or populations that were only transferred (squares). (**B**) The outcome of the experiments (n = 6) for the re-infected populations. Only in N2 there was a significant reduction in virus titer in the pre-exposed populations compared to the naive populations (ANOVA, P≤0.05). (**C**) The outcome of the experiment where the populations were transferred (so no re-infection). Also here only N2 showed a significant reduction in virus titer in the following generations compared to the first generation (ANOVA, P≤0.01).

In order to test this hypothesis an experiment was designed to determine the trans-generational effects of the RNAi response on the development of OrV infection in JU1580, N2 and both the *rde-2* and *rde-4* mutants (**Figure 4A**). In short, worms were synchronized and exposed to OrV at 26h (L2 stage). At 72h, infected worms were sampled and either transferred or bleached (to synchronize and get rid of OrV infection). The transferred worms were sampled again at 72h after transfer and the same process was repeated once more. The bleached group was re-infected at 26h and again sampled at 72h after bleaching. This cycle was also repeated for a third time. The worms in the transferred group are expected to show trans generational silencing effects, but not as severe as in the bleached group since the population is more mixed and contains the primary infected worms. However, if there is a strong negative effect in spread of the infection (as N2 needs a higher dose for establishing the infection), it will be seen in this group. In particular in the two RNAi mutants in the N2 background, these should then show lower infection levels independent of the RNAi effect. The bleached group will show the RNAi effect in particular as they are re-exposed to OrV every generation.

When the viral loads were compared upon OrV infection, only in N2 a significant difference in infection was found between the pre-exposed and naïve exposed populations (**Figure 4B and 4C** **, Figure S2**). A >10-fold decrease was found in the subsequent generations compared to the first generation (ANOVA, P≤0.05) (**Figure 4B**). Also in the transferred group there was a >3-fold decrease (ANOVA, P≤0.001) (**Figure 4C**). As expected, neither of the RNAi mutants (*rde-2, rde-4*) displayed a trans-generational effect of pre-exposure to OrV replication in the offspring, since no differential susceptibility to OrV infection was observed between naïve and pre-exposed worms. This result suggests that the effect seen in N2 is linked to the formation of a heritable RNAi response. Furthermore, since JU1580 does not show decreased replication of OrV in the offspring may indicate that this genotype cannot mount an effective heritable RNAi response.

## Discussion

The power of *C. elegans* as a model species initially prompted the development of artificial systems in which virus-host interactions could be studied in the worm. This research used vesicular stomatitis virus (VSV) and Flock house virus (FHV). Despite their wide host range, both viruses could only replicate within *C. elegans* by either using embryonic derived cells or using a transgenic system [19]–[21]. An important outcome from these studies was that the RNAi pathway had the capacity to limit viral replication via the involvement of the Argonaute RDE-1 [19]–[21], the nucleotidyltransferase MUT-2 [21], and the dsRNA binding protein RDE-4 [20], [21]. FHV was used to further identify genes involved in antiviral RNAi [25] and discover trans-generational inheritance of the antiviral small-interfering RNAs (viRNAs) [22]. The inheritance of an RNAi response was first established in the landmark paper by Fire *et al*. (1998), where gene silencing was induced in the progeny of worms injected with dsRNA [18]. Subsequent research identified the genes involved in initial silencing and in the transfer of the silencing response to the offspring [35], [36]. It became clear that the induction of siRNAs and inheritance of an RNAi response are different mechanisms [35] and that the inheritance requires formation of secondary siRNAs [21].

The *C. elegans* RNAi response is also important in limiting OrV replication, which was convincingly shown by experimental infection of a range of mutant *C. elegans* strains [7]. Also the phenotypic differences found between N2 and JU1580 have been linked to a polymorphism in the *drh-1* gene [23] and it seems that this gene is also involved in limiting OrV infection among wild isolates [24]. OrV persists in the natural JU1580 population due to efficient horizontal transmission from the infected worms to other worms and its offspring. To analyse the development of OrV infection with higher resolution we developed a quantitative infection assay. By infecting worm cohorts at different time points with OrV, progression of infection was monitored through quantification of viral RNA using qPCR. The influence of worm age and genotype on viral replication was determined using a defined viral dose for a defined incubation time.

In JU1580 larvae the mid-log phase was reached earlier in younger JU1580 larvae and coincided with a lower maximum infection load. These observations may be linked to the development of the intestine, the site of OrV infection [6], [7]. At the end of each larval stage the volume of the intestinal cells expand, as does the ploidity of the middle 10 intestinal cells [37]. Therefore, the maximum viral load measured could be limited by available space in the nematode. Next to that, the speed at which the infection develops is slower at older age. The cause of these observations remains unclear but could be elucidated by detailed immunofluorescence studies following the development of the infection.

In N2 there was a trend for higher susceptibility in younger larvae, but the effect was not as strong as in JU1580. The most striking difference was found in maximum viral load, which did not differ significantly between infections started in the respective larval stages in N2. This is in contrast with JU1580, where the maximum viral load increases with age. The reason for this could lie in a more limited infection in N2 [7] or a stronger antiviral response, persisting over time, to begin with. We found that in experiments carried out within one generation, where no offspring was present (infection at L1, L2 and L3 experiments), the differences between N2 and JU1580 were relatively small. However, when offspring was present (infection at L4 and the heritable RNAi experiment), we observed that the differences in viral load increased. The cause of this is unclear as it seems unlikely that larvae are already (highly) infected at this stage.

The difference in viral load phenotype of JU1580 and both the *rde-4* and *rde-2* mutants relative to N2 was small compared to the reported 100-fold differences in viral load by Félix *et al*. (2011) and prompted the hypothesis that trans-generational effects may play a role. Therefore, several subsequent generations exposed to virus were re-infected to determine if trans-generational effects could be observed in infections in *C. elegans* populations. These experiments showed that the RNAi response has a dual role in limiting infection; i) an RNAi response to limit OrV replication in the individual worm, combined with ii) a trans-generational effect rendering offspring of infected N2 less susceptible to viral replication. Consequently, N2 populations might lose the infection after a limited number of generations, whereas JU1580 populations remain infected.

This heritable RNAi response present in N2 appears absent or severely compromised in the wild isolate JU1580, which may be linked to the recently detected polymorphism in its *drh-1* gene [23], [24]. All the *C. elegans* strains that were isolated from the two sites where the *Caenorhabditis-*infecting viruses were found (Orsay and Santeuil) are polymorphic for *drh-1* [10]. *DRH-1* is a homologue of mammalian RIG-I and most likely a molecule with activity in the RNAi pathway involved in sensing non-self (e.g. viral) RNA [25], [26]. We found that the OrV infection develops faster in the *rde-4* mutant than in the *rde-2* and both are similar to JU1580. This, along with abnormalities of the small antiviral RNA response against OrV [7], [24], show that JU1580 cannot mount an effective early RNAi response. The finding that JU1580 does not show a trans-generational effect is indicative of an abrogated function in or upstream of secondary siRNA generation.

To conclude, we report a quantitative study of OrV replication and the discovery of trans-generational effects of antiviral RNAi. Dose-response analysis of different larval stages revealed that the progression speed of OrV infection decreased with subsequent larval stages (L1-L4) and higher maximum viral loads were reached in the older stages. Surprisingly, hitherto presumed OrV sensitive strain JU1580 showed similar susceptibility as N2 at exposure to higher viral doses in liquid inoculum. In contrast to JU1580, viral infection in N2 is controlled by a heritable RNAi response. Consequently, offspring of infected N2 is less susceptible to viral replication. We present a new quantitative infection assay using *C. elegans* which allows for studying the molecular details of OrV replication, thus facilitating virus-host interaction studies in a genetically tractable model organism.

## Supporting Information

Figure S1 **Logistic curve fits.** All the curve fits obtained for JU1580 (infected in L1, L2, L3 and L4), N2 (infected in L1, L2, L3 and L4), WM29 (*rde-2*, infected in L3) and WM49 (*rde-4*, infected in L3). The time is time post infection. Individual data points are shown in dots. Identified outliers are shown with an x instead of a dot. The sigmoidal curve fit +/− SD is shown in the dashed grey lines. The calculated inflection point and calculated asymptote are also shown. As is the R^2^ of the curve-fit.
(PDF)

Figure S2 **Heritable RNAi experiment.** All the individual data points for the heritable RNAi experiment (6 independent experiments) are shown, for the genotypes JU1580, N2, WM29 (*rde-2*) and WM49 (*rde-4*). The mean +/− SE are shown.
(PDF)

Table S1 **Reference genes** The mean Ct-values +/− SD for the reference genes per genotype and stage (NA means none available; indicates in which stages no experiments were done).
(PDF)

## References

[1] Kirienko NV, Mani K, Fay DS (2010) Cancer models in Caenorhabditis elegans. Dev Dyn 239: 1413–1448. 20175192 10.1002/dvdy.22247 PMC4098942

[2] Kenyon CJ (2010) The genetics of ageing. Nature 464: 504–512. 20336132 10.1038/nature08980

[3] Snoek LB, Sterken MG, Volkers RJ, Klatter M, Bosman KJ, et al (2014) A rapid and massive gene expression shift marking adolescent transition in C. elegans. Sci Rep 4: 3912. 24468752 10.1038/srep03912 PMC3904150

[4] Jager T, Alvarez OA, Kammenga JE, Kooijman SALM (2005) Modelling nematode life cycles using dynamic energy budgets. Functional Ecology 19: 136–144.

[5] Marsh EK, May RC (2012) Caenorhabditis elegans, a model organism for investigating immunity. Appl Environ Microbiol 78: 2075–2081. 22286994 10.1128/AEM.07486-11 PMC3302602

[6] Franz CJ, Renshaw H, Frezal L, Jiang Y, Félix M-A, et al (2014) Orsay, Santeuil and Le Blanc viruses primarily infect intestinal cells in Caenorhabditis nematodes. Virology 448: 255–264. 24314656 10.1016/j.virol.2013.09.024

[7] Felix MA, Ashe A, Piffaretti J, Wu G, Nuez I, et al (2011) Natural and experimental infection of Caenorhabditis nematodes by novel viruses related to nodaviruses. PLoS biology 9: e1000586. 21283608 10.1371/journal.pbio.1000586 PMC3026760

[8] Franz CJ, Zhao G, Felix MA, Wang D (2012) Complete genome sequence of Le Blanc virus, a third Caenorhabditis nematode-infecting virus. J Virol 86: 11940. 23043172 10.1128/JVI.02025-12 PMC3486331

[9] Andersen EC, Gerke JP, Shapiro JA, Crissman JR, Ghosh R, et al (2012) Chromosome-scale selective sweeps shape Caenorhabditis elegans genomic diversity. Nat Genet 44: 285–290. 22286215 10.1038/ng.1050 PMC3365839

[10] Volkers RJ, Snoek LB, van Hellenberg Hubar CJ, Coopman R, Chen W, et al (2013) Gene-environment and protein degradation signatures characterize genomic and phenotypic diversity in wild Caenorhabditis elegans populations. BMC Biol 11: 93. 23957880 10.1186/1741-7007-11-93 PMC3846632

[11] Kiontke KC, Felix MA, Ailion M, Rockman MV, Braendle C, et al (2011) A phylogeny and molecular barcodes for Caenorhabditis, with numerous new species from rotting fruits. BMC Evol Biol 11: 339. 22103856 10.1186/1471-2148-11-339 PMC3277298

[12] Grishkevich V, Ben-Elazar S, Hashimshony T, Schott DH, Hunter CP, et al (2012) A genomic bias for genotype-environment interactions in C. elegans. Mol Syst Biol 8: 587. 22669615 10.1038/msb.2012.19 PMC3397417

[13] Elvin M, Snoek LB, Frejno M, Klemstein U, Kammenga JE, et al (2011) A fitness assay for comparing RNAi effects across multiple C. elegans genotypes. BMC Genomics 12: 510. 22004469 10.1186/1471-2164-12-510 PMC3206879

[14] Felix MA, Braendle C (2010) The natural history of Caenorhabditis elegans. Curr Biol 20: R965–969. 21093785 10.1016/j.cub.2010.09.050

[15] Pujol N, Zugasti O, Wong D, Couillault C, Kurz CL, et al (2008) Anti-fungal innate immunity in C. elegans is enhanced by evolutionary diversification of antimicrobial peptides. PLoS Pathog 4: e1000105. 18636113 10.1371/journal.ppat.1000105 PMC2453101

[16] Kammenga JE, Phillips PC, De Bono M, Doroszuk A (2008) Beyond induced mutants: using worms to study natural variation in genetic pathways. Trends Genet 24: 178–185. 18325626 10.1016/j.tig.2008.01.001

[17] Viñuela A, Snoek LB, Riksen JAG, Kammenga JE (2010) Genome-wide gene expression regulation as a function of genotype and age in C-elegans. Genome Research 20: 929–937. 20488933 10.1101/gr.102160.109 PMC2892094

[18] Fire A, Xu S, Montgomery MK, Kostas SA, Driver SE, et al (1998) Potent and specific genetic interference by double-stranded RNA in Caenorhabditis elegans. Nature 391: 806–811. 9486653 10.1038/35888

[19] Lu R, Maduro M, Li F, Li HW, Broitman-Maduro G, et al (2005) Animal virus replication and RNAi-mediated antiviral silencing in Caenorhabditis elegans. Nature 436: 1040–1043. 16107851 10.1038/nature03870 PMC1388260

[20] Wilkins C, Dishongh R, Moore SC, Whitt MA, Chow M, et al (2005) RNA interference is an antiviral defence mechanism in Caenorhabditis elegans. Nature 436: 1044–1047. 16107852 10.1038/nature03957

[21] Schott DH, Cureton DK, Whelan SP, Hunter CP (2005) An antiviral role for the RNA interference machinery in Caenorhabditis elegans. Proc Natl Acad Sci U S A 102: 18420–18424. 16339901 10.1073/pnas.0507123102 PMC1317933

[22] Rechavi O, Minevich G, Hobert O (2011) Transgenerational inheritance of an acquired small RNA-based antiviral response in C. elegans. Cell 147: 1248–1256. 22119442 10.1016/j.cell.2011.10.042 PMC3250924

[23] Sarkies P, Ashe A, Le Pen J, McKie MA, Miska EA (2013) Competition between virus-derived and endogenous small RNAs regulates gene expression in Caenorhabditis elegans. Genome Res 23: 1258–1270. 23811144 10.1101/gr.153296.112 PMC3730100

[24] Ashe A, Belicard T, Le Pen J, Sarkies P, Frezal L, et al (2013) A deletion polymorphism in the Caenorhabditis elegans RIG-I homolog disables viral RNA dicing and antiviral immunity. Elife 2: e00994. 24137537 10.7554/eLife.00994 PMC3793227

[25] Lu R, Yigit E, Li WX, Ding SW (2009) An RIG-I-Like RNA helicase mediates antiviral RNAi downstream of viral siRNA biogenesis in Caenorhabditis elegans. PLoS Pathog 5: e1000286. 19197349 10.1371/journal.ppat.1000286 PMC2629121

[26] Guo XY, Zhang R, Wang J, Ding SW, Lu R (2013) Homologous RIG-I-like helicase proteins direct RNAi-mediated antiviral immunity in C. elegans by distinct mechanisms. Proceedings of the National Academy of Sciences of the United States of America 110: 16085–16090. 24043766 10.1073/pnas.1307453110 PMC3791698

[27] Brenner S (1974) Genetics of Caenorhabditis-Elegans. Genetics 77: 71–94. 4366476 10.1093/genetics/77.1.71 PMC1213120

[28] Emmons SW, Klass MR, Hirsh D (1979) Analysis of the Constancy of DNA Sequences during Development and Evolution of the Nematode Caenorhabditis-Elegans. Proc Natl Acad Sci U S A 76: 1333–1337. 286315 10.1073/pnas.76.3.1333 PMC383245

[29] Le Novere N (2001) MELTING, computing the melting temperature of nucleic acid duplex. Bioinformatics 17: 1226–1227. 11751232 10.1093/bioinformatics/17.12.1226

[30] Bates DM, Chambers JM (1992) Nonlinear models. In: Chambers JM, Hastie TJ, editors. Statistical Models in S. London: WAdsworth & Brooks/Cole.

[31] Tijsterman M, Ketting RF, Okihara KL, Sijen T, Plasterk RH (2002) RNA helicase MUT-14-dependent gene silencing triggered in C. elegans by short antisense RNAs. Science 295: 694–697. 11809977 10.1126/science.1067534

[32] Sundaram P, Han W, Cohen N, Echalier B, Albin J, et al (2008) Caenorhabditis elegans ABCRNAi transporters interact genetically with rde-2 and mut-7. Genetics 178: 801–814. 18245353 10.1534/genetics.107.081588 PMC2248369

[33] Blanchard D, Parameswaran P, Lopez-Molina J, Gent J, Saynuk JF, et al (2011) On the nature of in vivo requirements for rde-4 in RNAi and developmental pathways in C. elegans. RNA Biology 8: 458–467. 21519199 10.4161/rna.8.3.14657 PMC3218512

[34] Parrish S, Fire A (2001) Distinct roles for RDE-1 and RDE-4 during RNA interference in Caenorhabditis elegans. RNA 7: 1397–1402. 11680844 PMC1370183

[35] Grishok A (2000) Genetic Requirements for Inheritance of RNAi in C. elegans. Science 287: 2494–2497. 10741970 10.1126/science.287.5462.2494

[36] Vastenhouw NL, Brunschwig K, Okihara KL, Muller F, Tijsterman M, et al (2006) Gene expression: long-term gene silencing by RNAi. Nature 442: 882. 16929289 10.1038/442882a

[37] Altun ZF, Hall DH (2009) Alimentary system, intestine. In: Herndon LA, editor.

