# Supplementary figures and images for: A Heritable Antiviral RNAi Response Limits Orsay Virus Infection in Caenorhabditis elegans N2
Source: PLoS One. 2014 Feb 24;9(2):e89760. doi: 10.1371/journal.pone.0089760 (PMC3933659; doi:10.1371/journal.pone.0089760)

# JU1580 L1

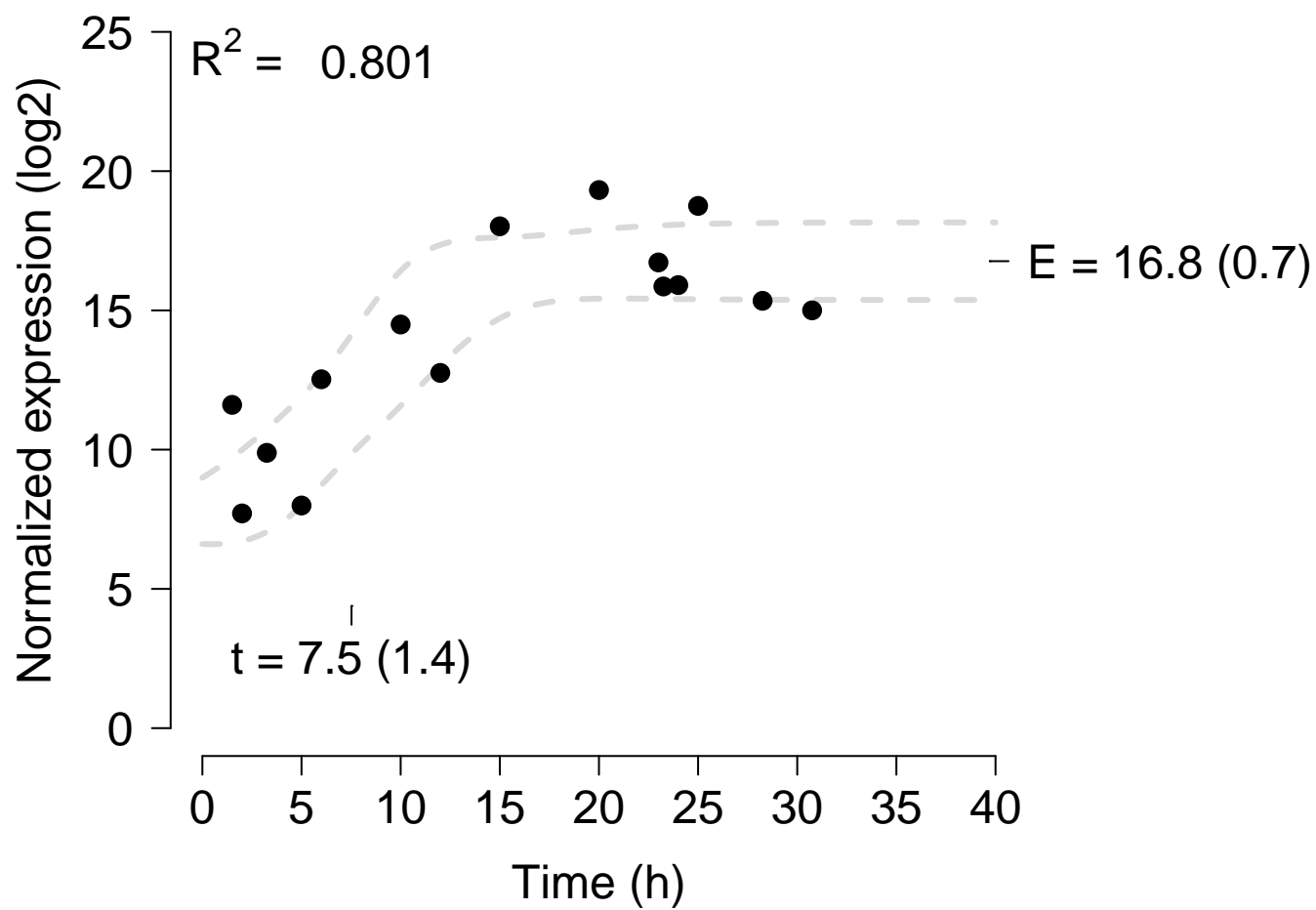

# JU1580 L2

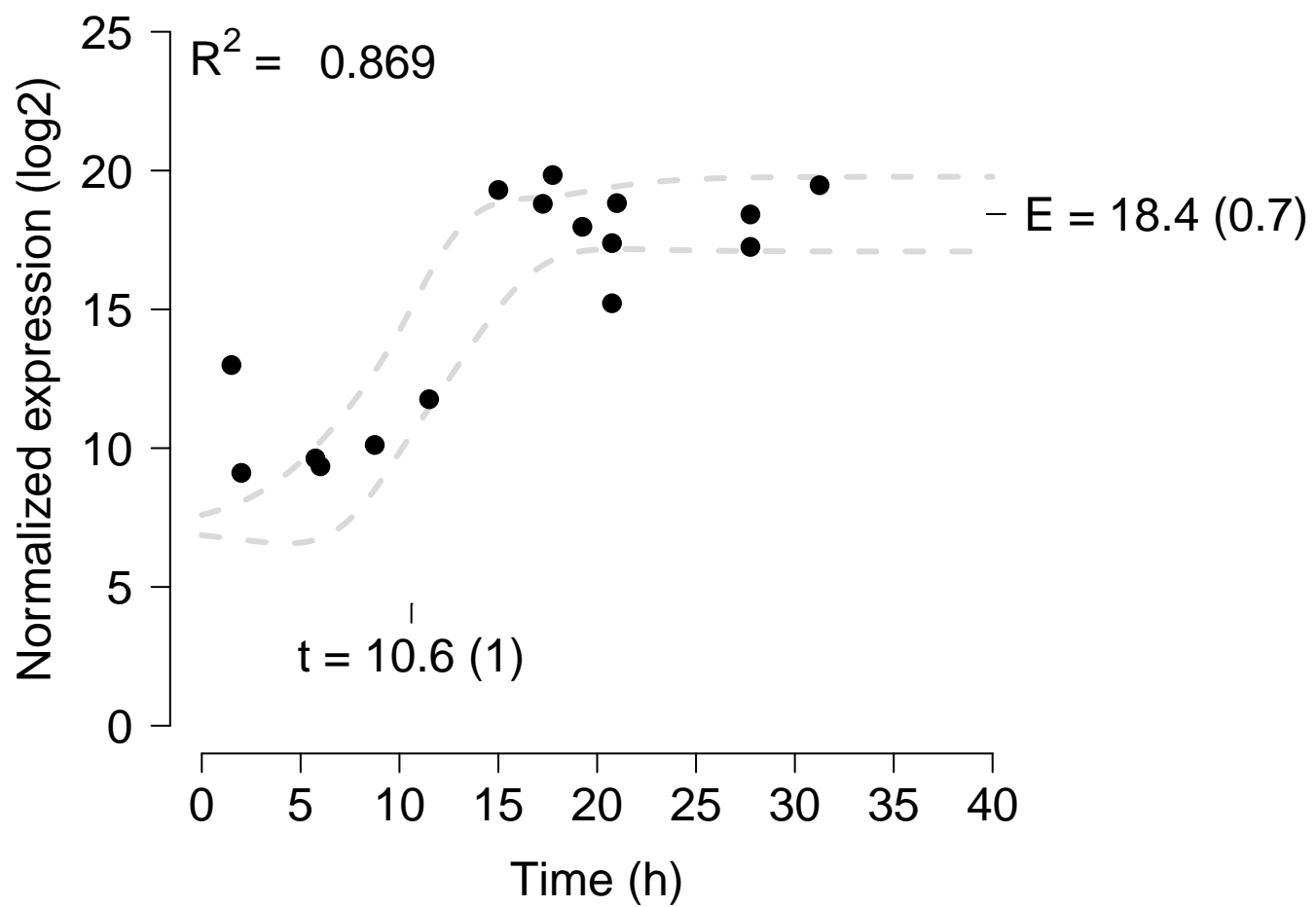

# JU1580 L3

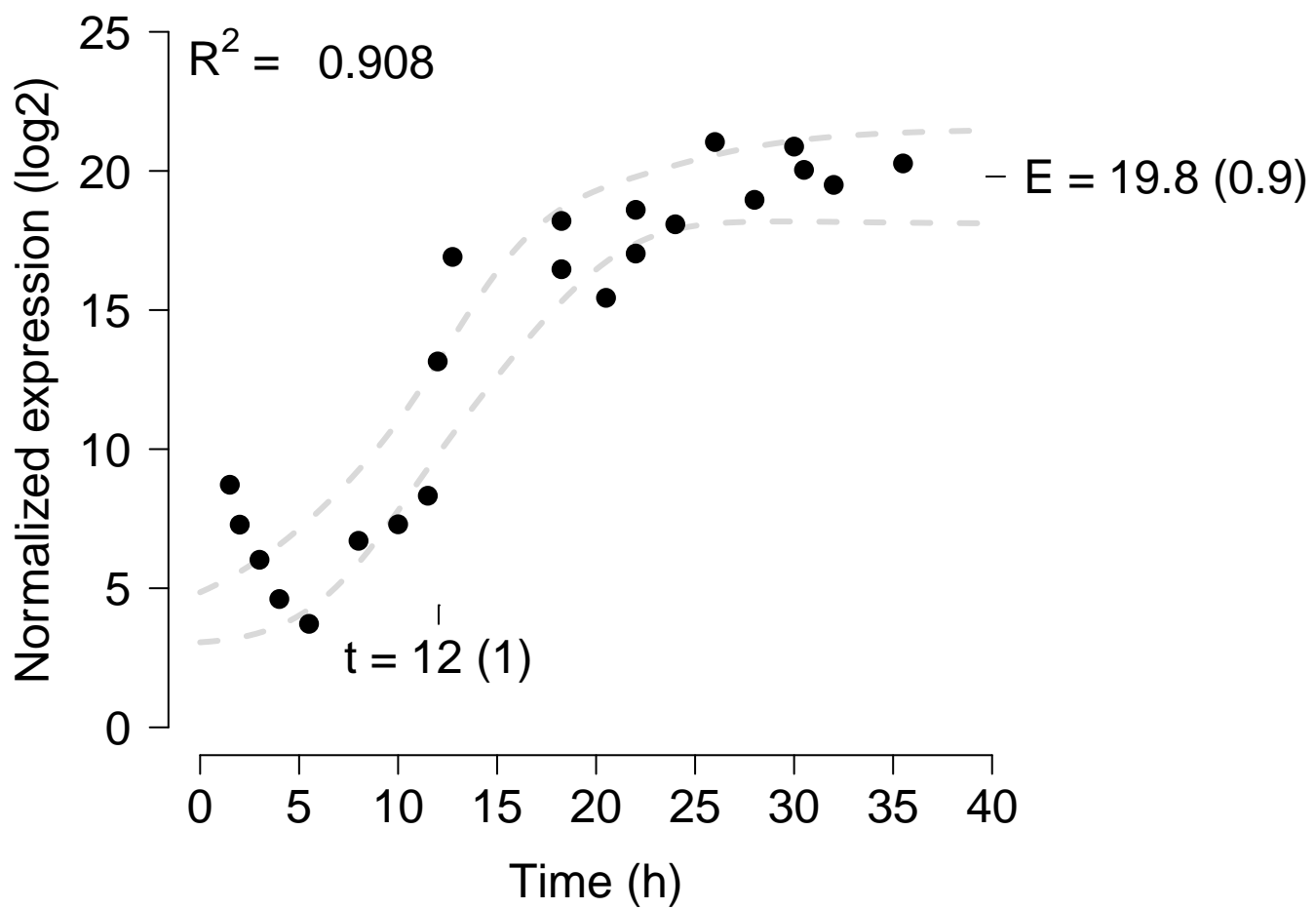

# JU1580 L4

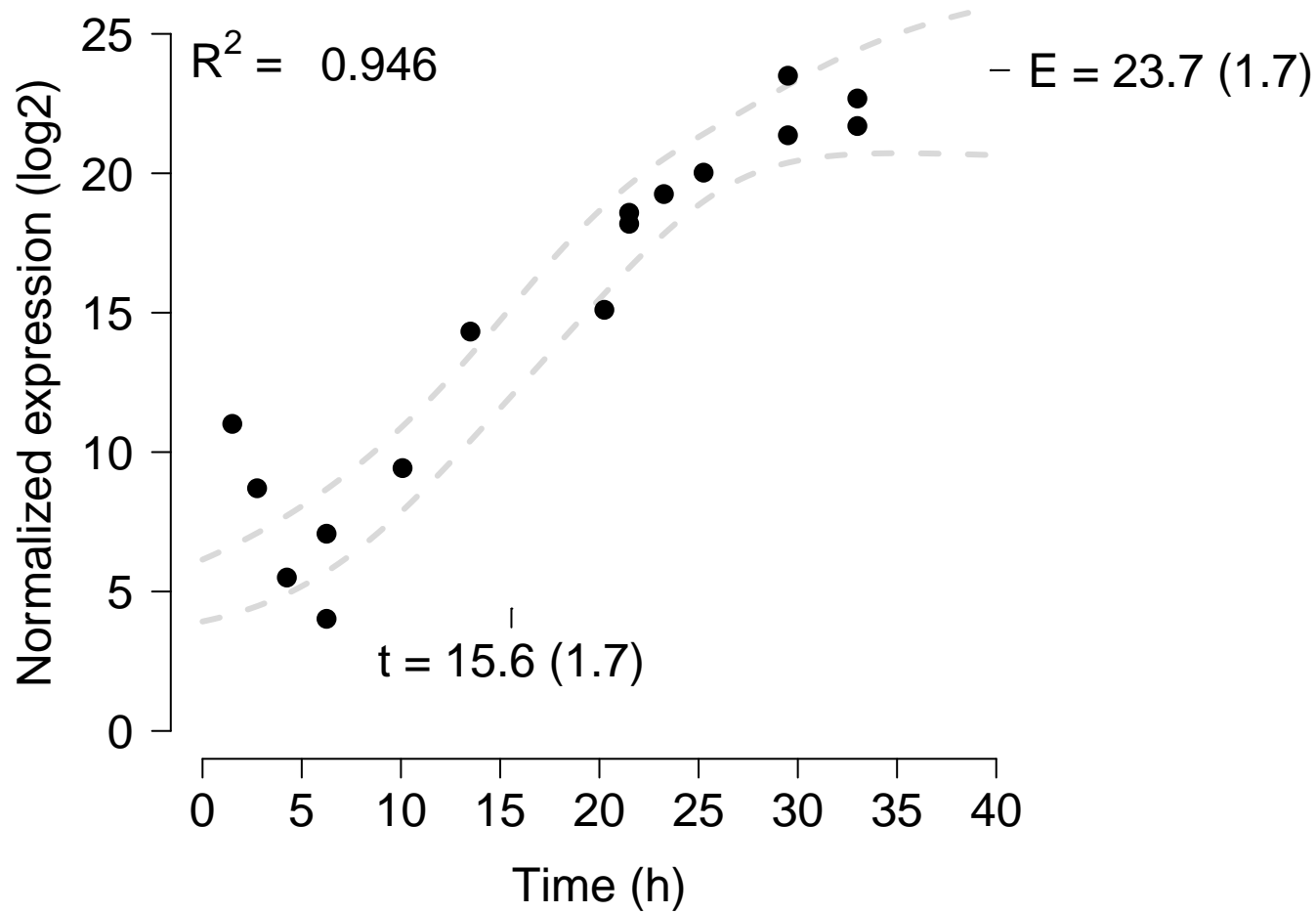

# N2 L1

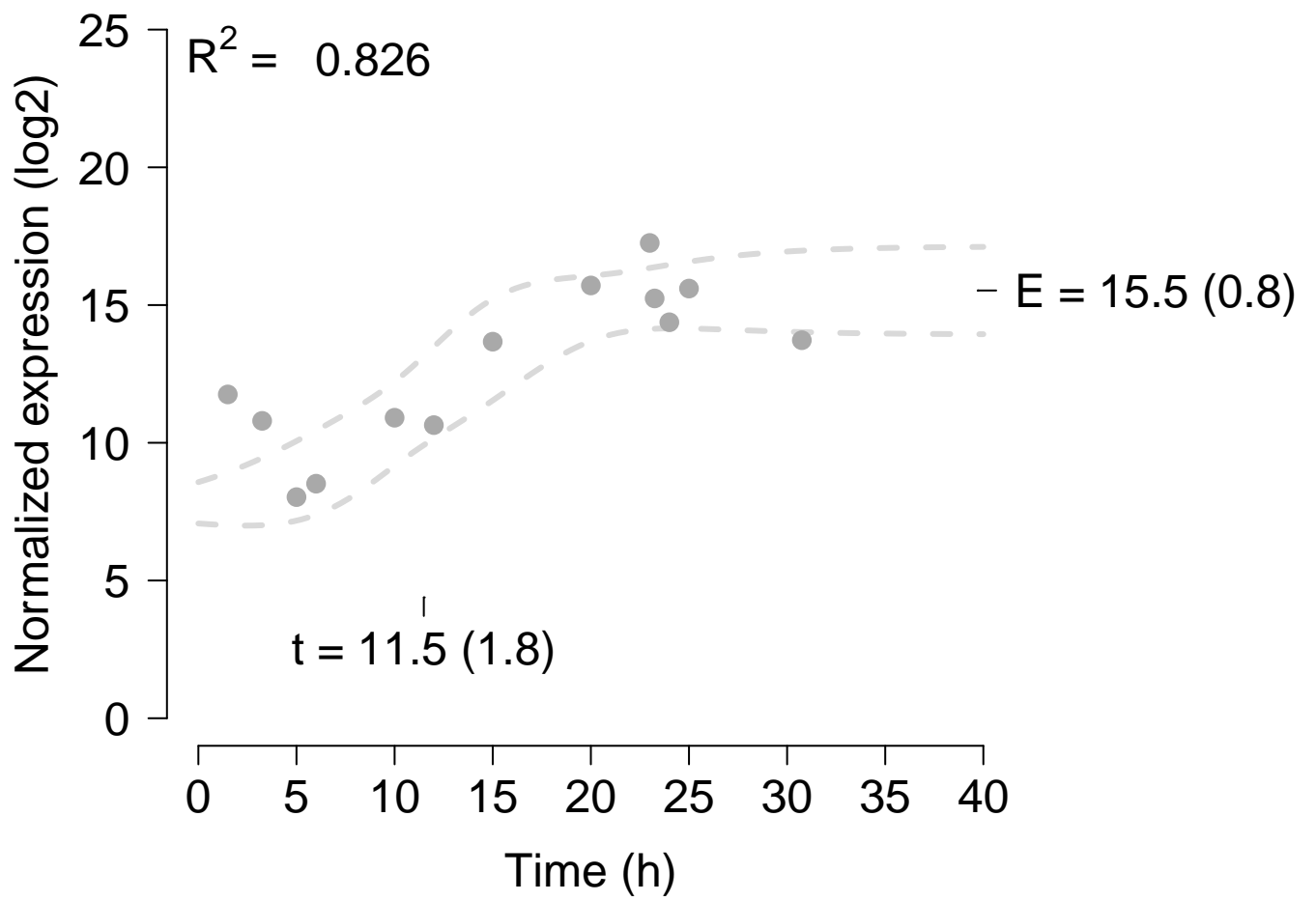

## N2 L2

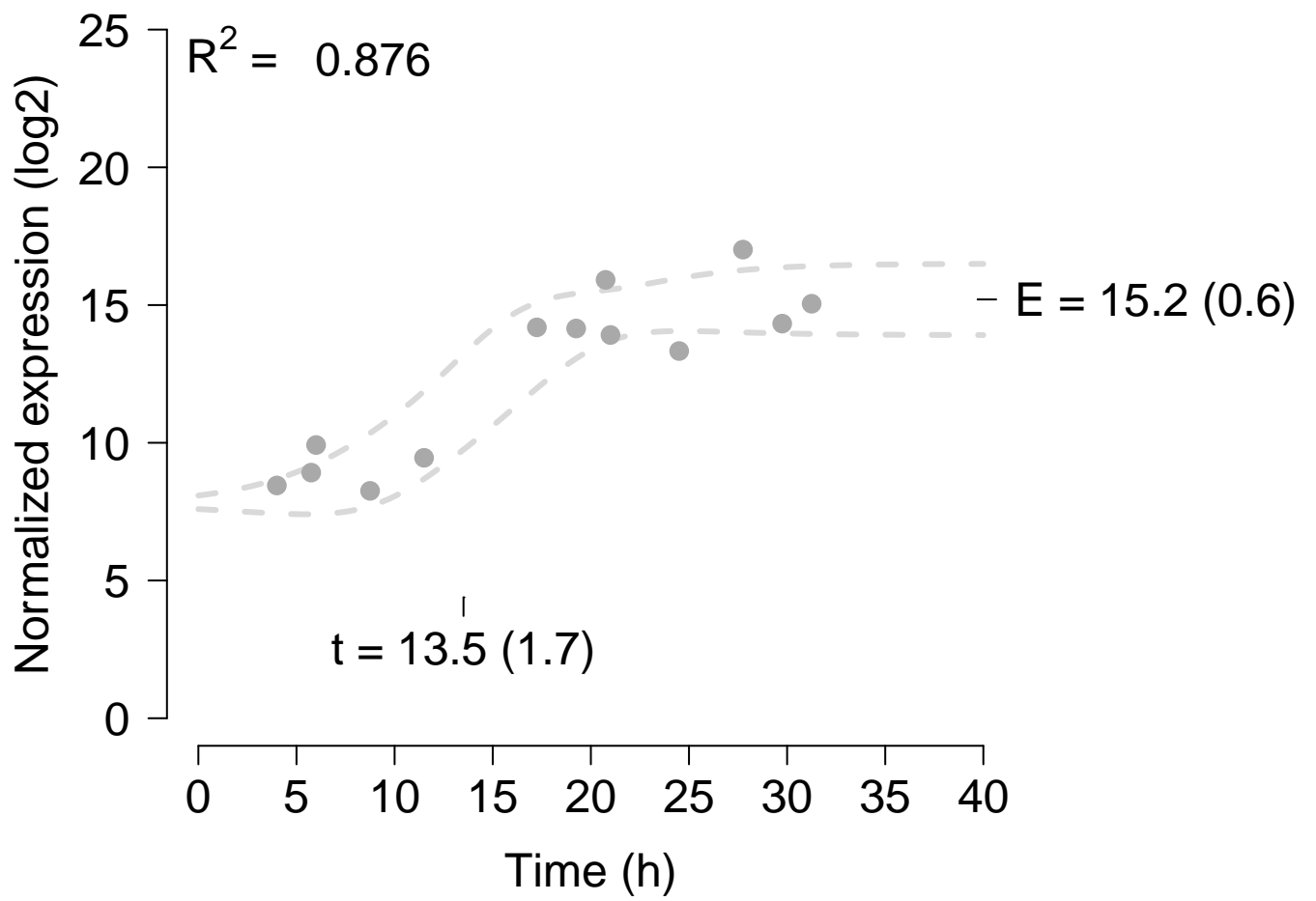

# N2 L3

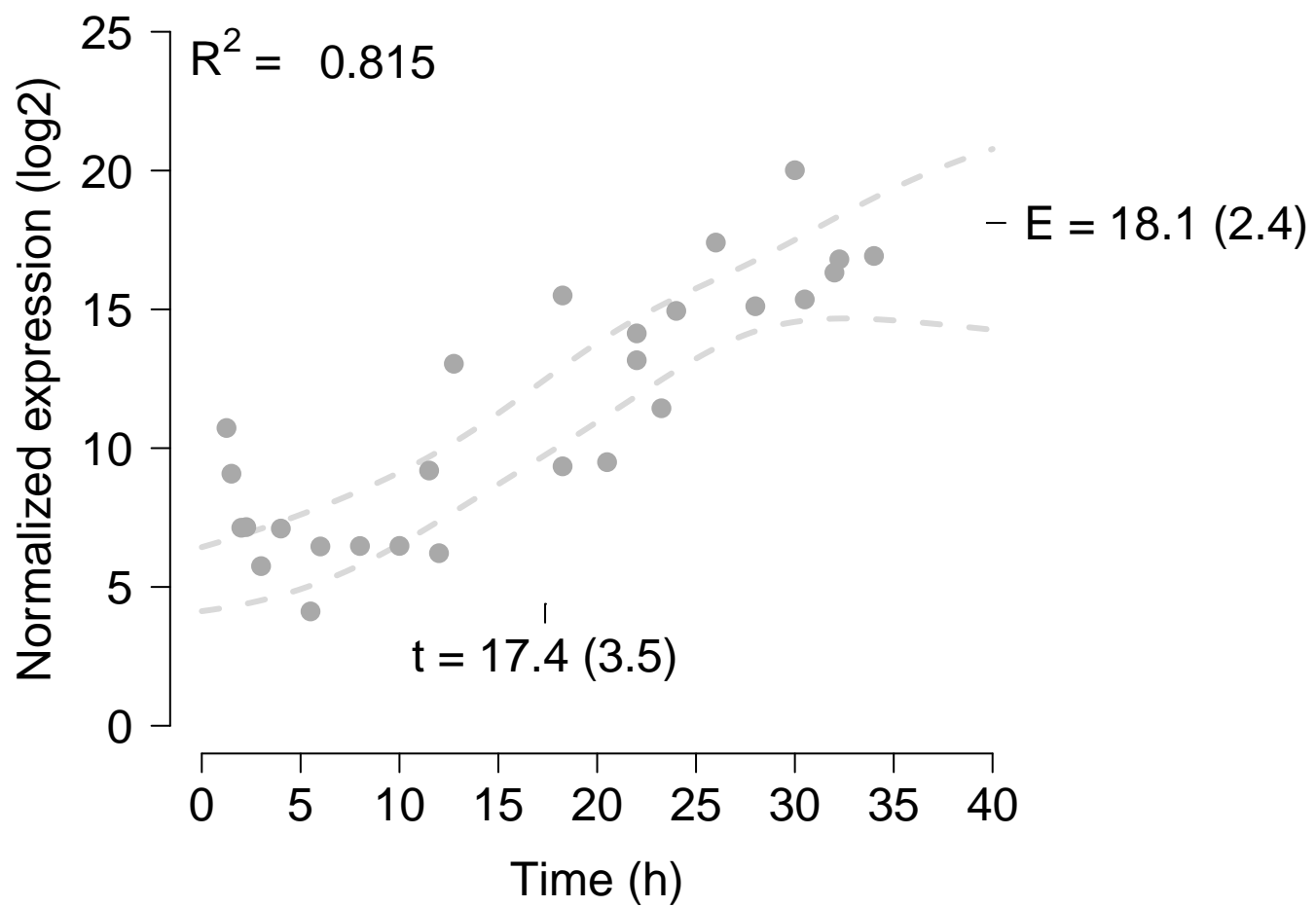

# N2 L4

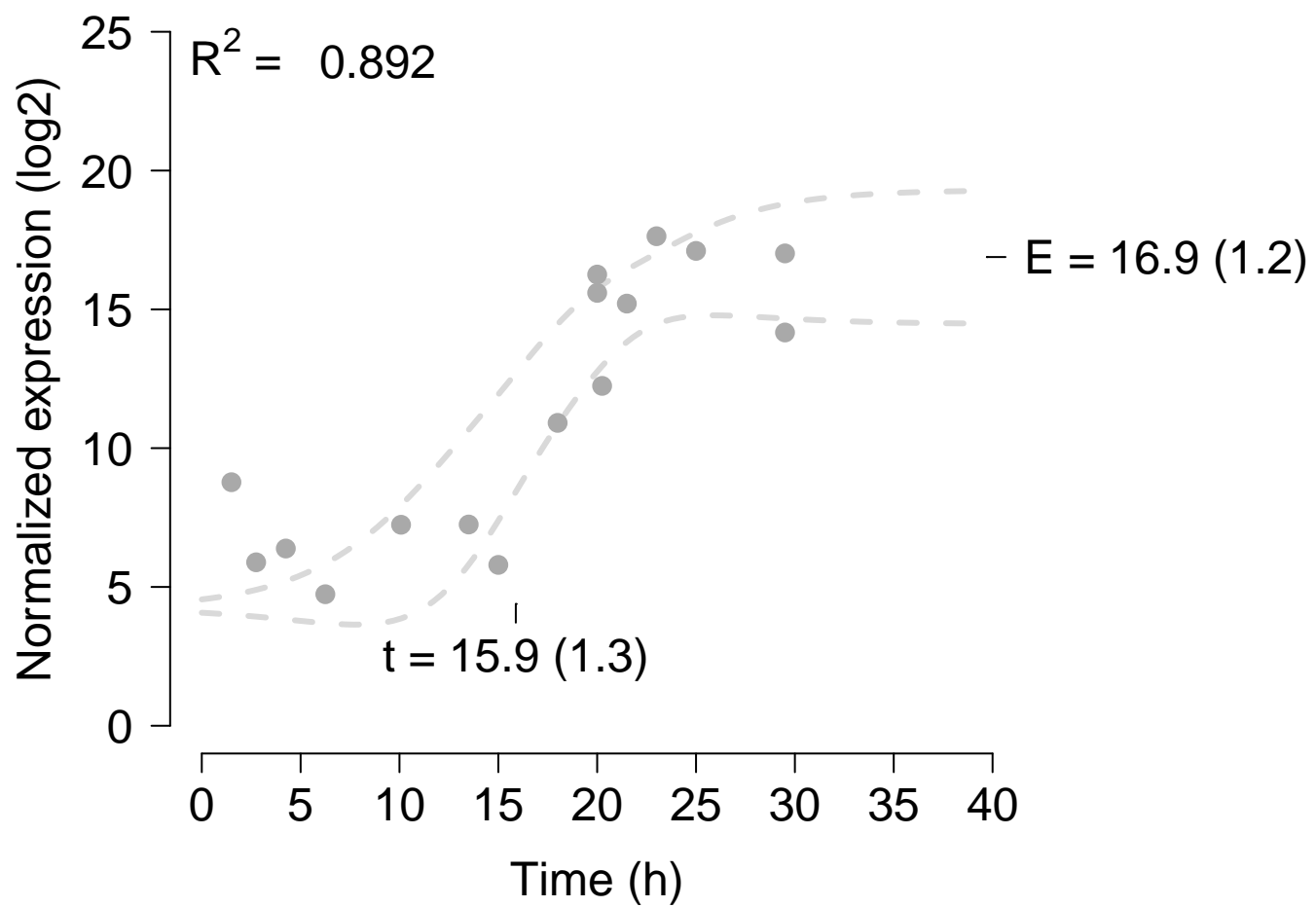

# rde-2 L3

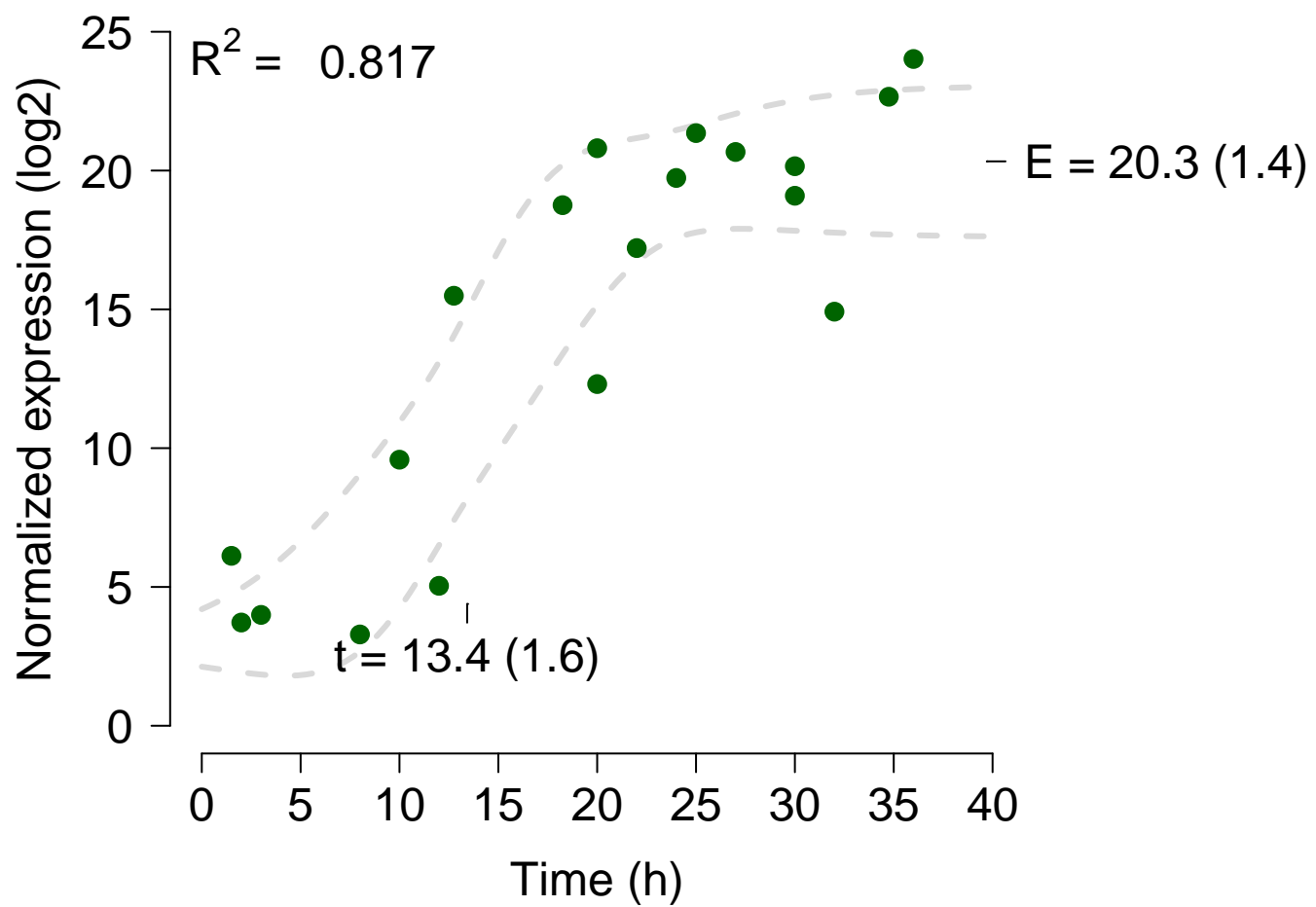

# rde-4 L3

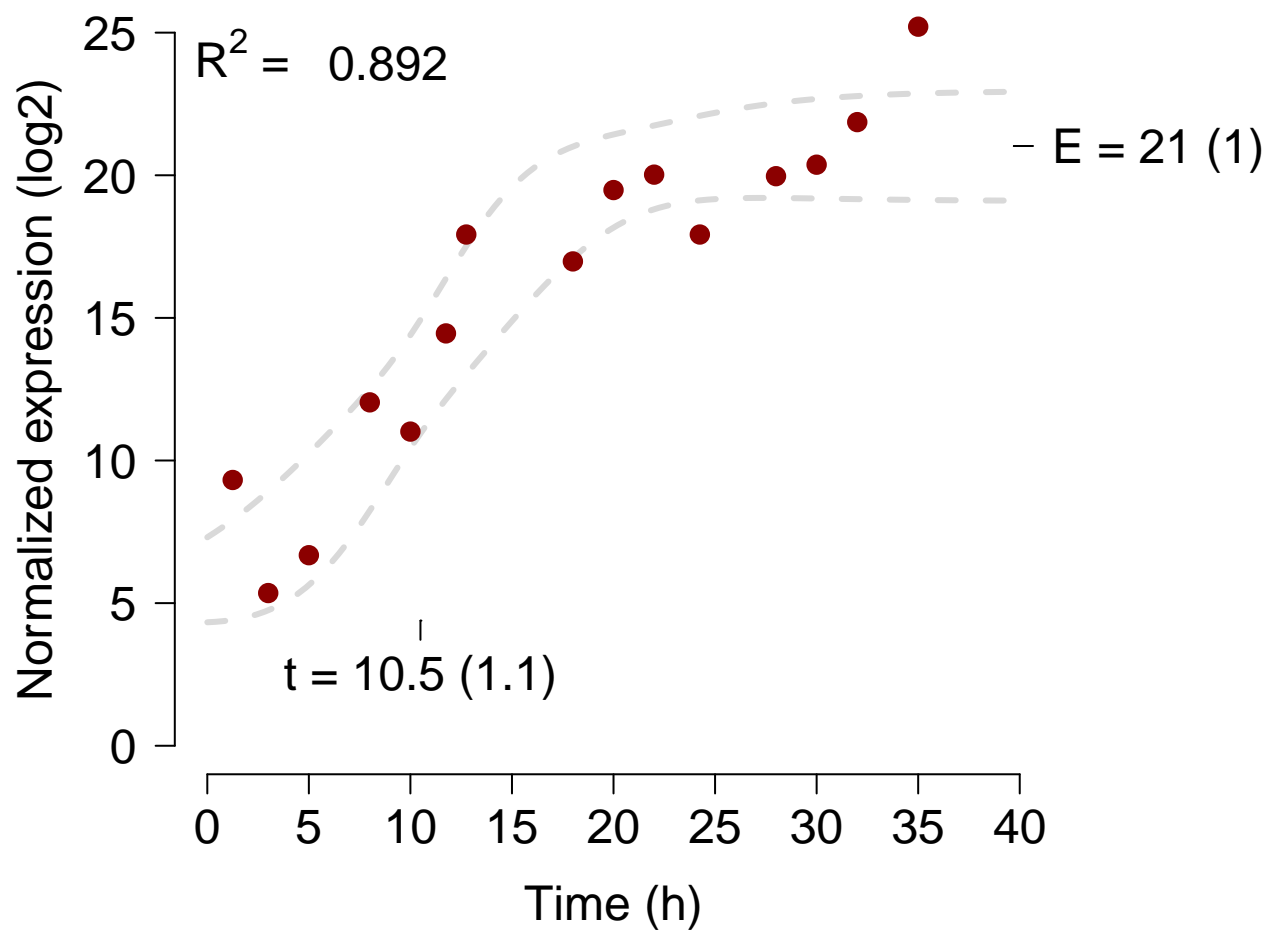

Supplement: Figure S1 — Logistic curve fits. All the curve fits obtained for JU1580 (infected in L1, L2, L3 and L4), N2 (infected in L1, L2, L3 and L4), WM29 (rde-2, infected in L3) and WM49 (rde-4, infected in L3). The time is time post infection. Individual data points are shown in dots. Identified outliers are shown with an x instead of a dot. The sigmoidal curve fit +/− SD is shown in the dashed grey lines. The calculated inflection point and calculated asymptote are also shown. As is the R2 of the curve-fit. (PDF) [file pone.0089760.s001.pdf]

N2 control

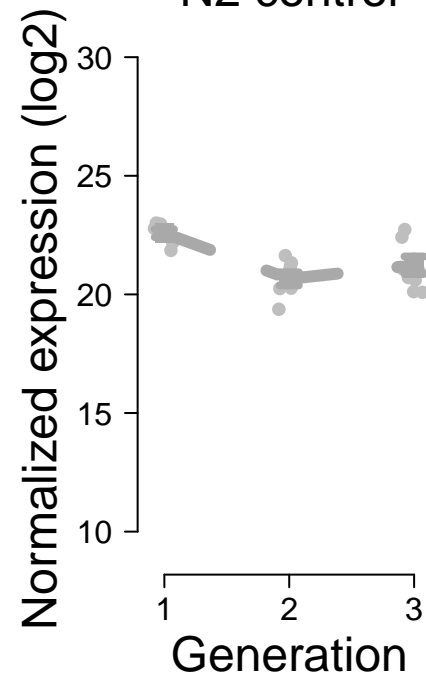

N2 bleach

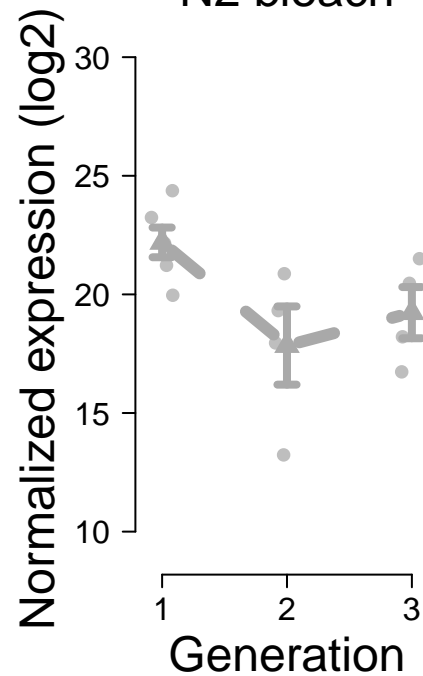

JU1580 control

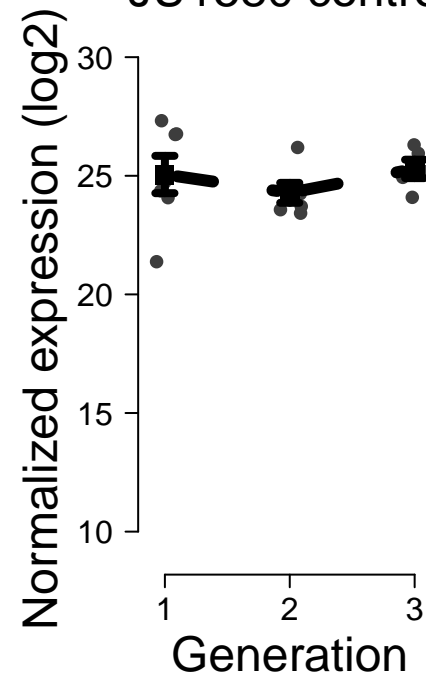

JU1580 bleach

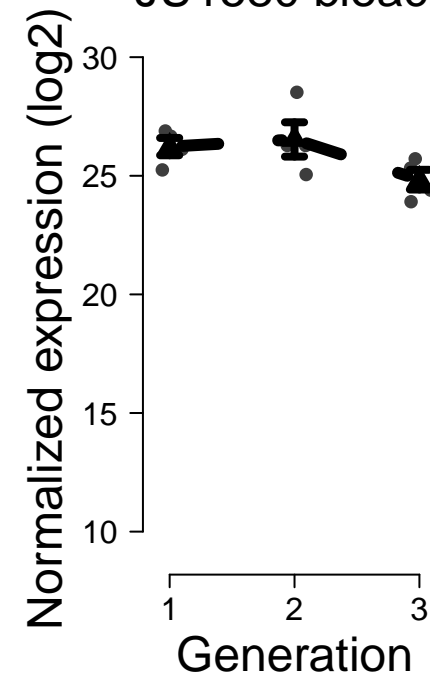

WM29 control

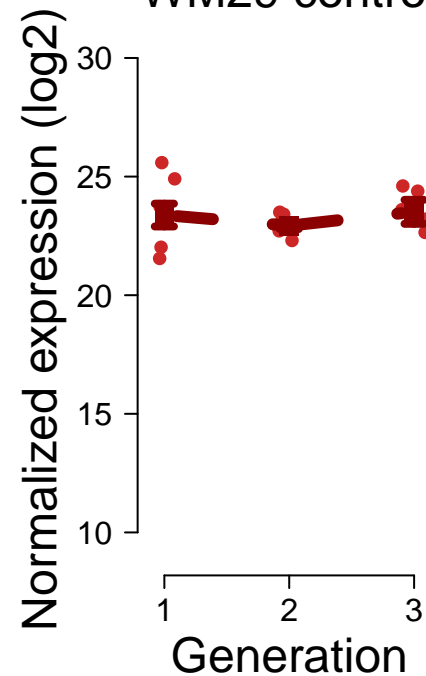

WM29 bleach

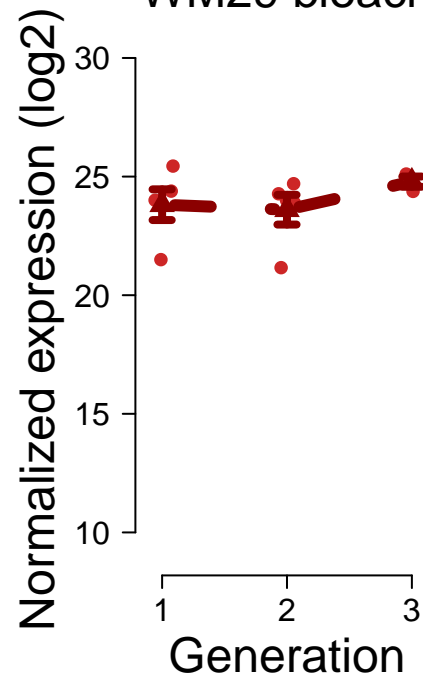

WM49 control

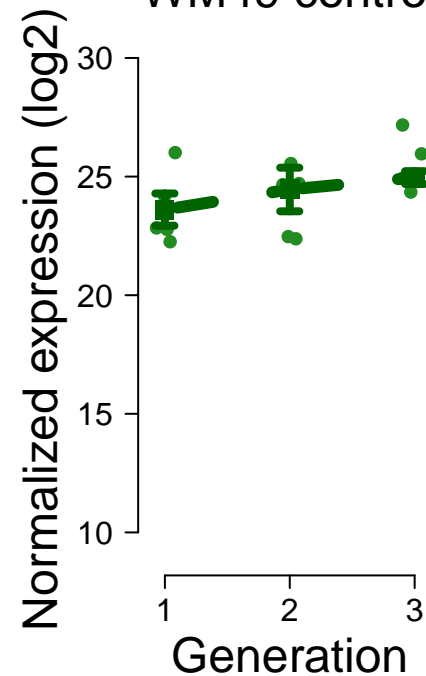

WM49 bleach

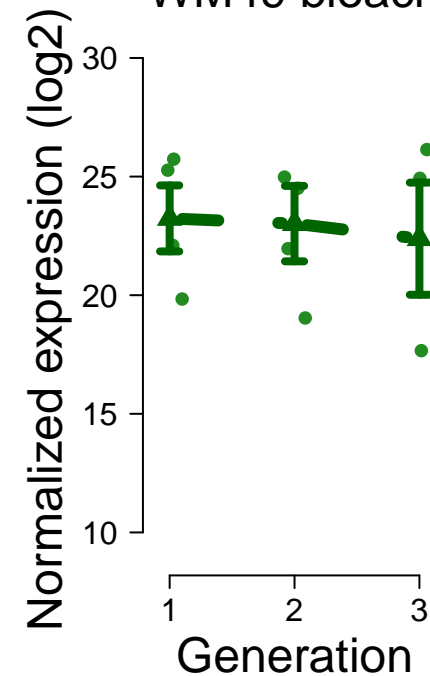

Supplement: Figure S2 — Heritable RNAi experiment. All the individual data points for the heritable RNAi experiment (6 independent experiments) are shown, for the genotypes JU1580, N2, WM29 (rde-2) and WM49 (rde-4). The mean +/− SE are shown. (PDF) [file pone.0089760.s002.pdf]
