# Supplementary material for: A Heritable Antiviral RNAi Response Limits Orsay Virus Infection in Caenorhabditis elegans N2
Source: PLoS One. 2014 Feb 24;9(2):e89760. doi: 10.1371/journal.pone.0089760 (PMC3933659; doi:10.1371/journal.pone.0089760)
Supplement: Table S1 — Reference genes The mean Ct-values +/− SD for the reference genes per genotype and stage (NA means none available; indicates in which stages no experiments were done). (PDF) [file pone.0089760.s003.pdf]

Gene	strain	L1	L2	L3	L4	mixed
Y37E3.7	JU1580	22.6 (1.63)	22.4 (1.51)	21.6 (2.33)	23.5 (2.21)	21.4 (2)
	N2	22.2 (1.54)	22 (1.24)	21.6 (2.24)	24 (1.85)	21.4 (1.47)
	WM29	NA	NA	22.5 (1.09)	NA	NA
	WM49	NA	NA	22.4 (0.82)	NA	NA
rpl-6	JU1580	22.9 (2.03)	21.4 (1.73)	20.5 (2.81)	22.3 (1.85)	20.1 (1.84)
	N2	22 (1.93)	20.8 (1.27)	20.3 (2.41)	22.4 (1.79)	20.6 (1.57)
	WM29	NA	NA	20.3 (1.14)	NA	NA
	WM49	NA	NA	20.7 (0.81)	NA	NA
